# Simulating structural plasticity of large scale networks in NEST

**DOI:** 10.1186/1471-2202-15-S1-P194

**Published:** 2014-07-21

**Authors:** Mikael Naveau, Markus Butz-Ostendorf

**Affiliations:** 1 Simulation Lab Neuroscience - Bernstein Facility for Simulation and Database Technology, Institute for Advanced Simulation, Jülich Aachen Research Alliance, Forschungszentrum Jülich, 52425 Jülich, Germany

## 

The brain is much less hard-wired as traditionally thought. Permanently, new synapses are formed, existing synapses are deleted or connectivity rewires by re-routing axonal branches (structural plasticity). However, all current large-scale neuronal network models are hard-wired with plasticity merely arising from changes in the strength of existing synapses, therefore missing an important aspect of the plasticity of brain networks. This project is to develop the first large-scale neuronal network model with structural plasticity in the neuronal network simulator NEST [1] and to make it scalable for HPC.

**Figure 1:**
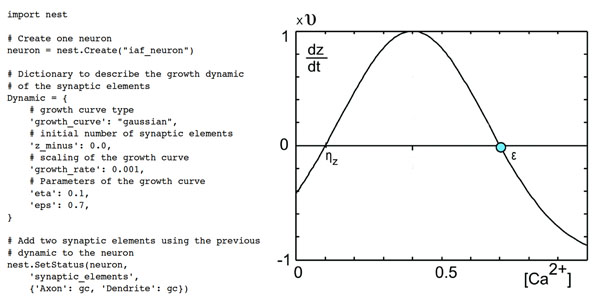
Example of the definition of two synaptic elements for an integrate and fire neuron using PyNEST. Here, the growth curve of the synaptic element is a Gaussian defined by three parameters: υ (the growth rate), η_z_ and ε (intersections with the x-axis). The growth dynamic depends on the electrical activity of the neuron modeled by the intracellular concentration of calcium.

Formation and deletion of synapses in the model for structural plasticity (MSP) [2] depends on the number of synaptic contact possibilities that each neuron has, i.e. the number of axonal boutons and dendritic spines. Therefore, we developed a framework that allows the addition of synaptic elements (i.e. axonal boutons or dendritic spines) for every neuron model already implemented in NEST. The user can then define its own synaptic elements and their corresponding growth dynamic depending on the electrical activity (see Figure 1). Synapses are formed by merging corresponding synaptic elements or are deleted when synaptic elements are lost. The update in connectivity depends on the availability of the synaptic elements in the entire networks. To make this model scalable for HPC, we developed a probabilistic approach that reduce both communication between compute nodes and their memory usage.

This implementation of the MSP in NEST allows neuroscientists to address important scientific questions on how large-scale networks rewire their connectivity in response to distortions in electrical activity balances
